# Standardized Application of Laxatives and Physical Measures in Neurosurgical Intensive Care Patients Improves Defecation Pattern but Is Not Associated with Lower Intracranial Pressure

**DOI:** 10.1155/2014/367251

**Published:** 2014-12-31

**Authors:** Martin Kieninger, Barbara Sinner, Bernhard Graf, Astrid Grassold, Sylvia Bele, Milena Seemann, Holger Künzig, Nina Zech

**Affiliations:** ^1^Department of Anesthesiology, University Hospital Regensburg, Franz-Josef-Strauß-Allee 11, 93053 Regensburg, Germany; ^2^Department of Neurosurgery, University Hospital Regensburg, Franz-Josef-Strauß-Allee 11, 93053 Regensburg, Germany

## Abstract

*Background*. Inadequate bowel movements might be associated with an increase in intracranial pressure in neurosurgical patients. In this study we investigated the influence of a structured application of laxatives and physical measures following a strict standard operating procedure (SOP) on bowel movement, intracranial pressure (ICP), and length of hospital stay in patients with a serious acute cerebral disorder.* Methods*. After the implementation of the SOP patients suffering from a neurosurgical disorder received pharmacological and nonpharmacological measures to improve bowel movements in a standardized manner within the first 5 days after admission to the intensive care unit (ICU) starting on day of admission. We compared mean ICP levels, length of ICU stay, and mechanical ventilation to a historical control group. *Results*. Patients of the intervention group showed an adequate defecation pattern significantly more often than the patients of the control group. However, this was not associated with lower ICP values, fewer days of mechanical ventilation, or earlier discharge from ICU. *Conclusions*. The implementation of a SOP for bowel movement increases the frequency of adequate bowel movements in neurosurgical critical care patients. However, this seems not to be associated with reduced ICP values.

## 1. Introduction

Motility disorders and disturbed defecation pattern are an important everyday challenge in intensive care practice. Prolonged periods of analgosedation, as it is often necessary after traumatic brain injury (TBI) or subarachnoid hemorrhage (SAH), may lead to insufficient bowel movement and even a state resembling paralytic ileus. This can lead to an increase in intra-abdominal pressure (IAP) and potentially result in an intracranial pressure (ICP) elevation [1]. In addition, an insufficient defecation rate is possibly associated with prolonged ventilation, increased rate of bacterial infection, and a higher mortality rate [2–8]. However, high-rate or high-volume defecations result in increased nursing efforts and potentially an increased patient risk due to dehydration [9, 10] and more frequent positioning maneuvers that could subsequently lead to increased ICP especially in patients with acute brain injury.

Until now no consistent regime has been implemented to optimize defecation patterns in ICU patients, especially in neurosurgical patients where it might be of advantage to have controlled defecation patterns. Therefore a standard operating procedure (SOP) dealing with the stimulation of bowel movements and the regulation of defecation pattern was implemented in our 10-bed neurointensive care unit. This SOP included drug-based as well as non-drug-related procedures for patients with acute severe cerebral disorder with expected prolonged sedation and ventilation need.

The aim of the present preliminary study with before-and-after design was to investigate if the implementation of a SOP concerning the stimulation of bowel movements has the ability to improve regularity of defecation pattern in patients with acute severe cerebral injuries. Furthermore we wanted to assess the influence of this SOP on ICP levels, ventilator dependency, and the length of ICU stay. The results of the intervention group were compared with a historical control group, in which promotility drugs were given only following clinical assessment and the attending physician's estimation of the patient's condition.

## 2. Methods

The study was approved by the ethical care committee of the University of Regensburg (approval number 12-101-0240).

We implemented the promotility bundle consisting of a daily increasing combination of pharmacological and nonpharmacological interventions for 5 days starting on day of admission (for details see Table 1) in our ICU on May 1, 2012. Criteria for being treated due to the SOP were age older than 18 years, a severe brain injury such as TBI, SAH, or intracerebral hemorrhage (ICH), or other serious cerebral disorders with need for continuous ICP monitoring and that continuing analgosedation and invasive ventilation for more than three days was estimated to be necessary by the attending physician on admission. Exclusion criteria were age under 18 years, pregnancy, severe gastrointestinal diseases, no ICP monitoring, no brain injury, or estimated shorter periods of analgosedation.

The SOP was stored in our PDMS (MetaVision Suite, iMDsoft), and once activated it always had the same sequence as stated in Table 1. The ICU staff was informed about the SOP and the handling of the intervention bundle prior to implementation.

After implementation of the SOP, patients meeting the inclusion criteria were consecutively treated with the intervention bundle between May 2012 and June 2013. In a before-and-after study, this “intervention group” was then compared with a historical patient population receiving promotility drugs in a nonstandardized fashion. This control group was recruited out of patients who had been admitted to our ICU before implementation of the SOP between August 2008 and April 2012. All patients older than 18 years with acute serious cerebral disorder and excluded pregnancy who had needed analgosedation and invasive ventilation for more than three days as well as invasive measurement of ICP were included.

For analgosedation, propofol, ketamine, midazolam, and sufentanil were used in both groups, dose-tailored to the assessment of the attending intensivist. For circulation maintenance norepinephrine was used as first-line vasopressor. The vasopressor dose was adjusted individually to maintain adequate cerebral perfusion pressure (CPP) for each patient. Nutritional support was given according to the guidelines of the German Society for Nutrition (DGEM 2007, http://www.dgem.de/) and the European Society for Clinical Nutrition and Metabolism (ESPEN 2005; http://www.espen.org/). Depending on the residual gastric volume of the previous day, the amount of ordered enteral nutrition was either reduced or retained or increased (up to maximum target volume) on the following day. In all included patients ICP was continuously monitored using the Neurovent ICP probe by Raumedic.

For each study day the following data were extracted from the PDMS: gut motility parameters (defecation frequency, stool volume, and consistency), applied promotility drugs (dosage and ordering schedule), nonpharmacological promotility procedures, analgosedation and circulatory-support parameters (mean daily dosage), ICP levels (mean daily values), daily amount of enteral nutrition, and fluid balances. In addition, duration of mechanical ventilation and length of ICU stay were documented.

The defecation pattern was recorded separately for each of the 5 study days (0:00 a.m. to 11:59 p.m.). A cumulative stool volume of more than 150 mL/24 and less than 500 mL/24 was defined as adequate, and a cumulative stool volume of less than 150 mL per day was insufficient. More than 500 mL per day, liquid stool consistency, or a daily defecation frequency of more than 3 was judged excessive. According to these criteria, patients were divided into two groups: group 1 (“adequate defecation”) consisted of patients, whose defecation pattern was adequate for more than one day within the study period and who never showed excessive defecation. Group 2 (“inadequate defecation”) included all patients, whose defecation patterns were insufficient or excessive, respectively. Additionally patients with at least one day of excessive defecation were recorded separately.

Statistical analysis was done using IBM SPSS Statistics Version 21. Categorical data were displayed according to their distribution frequency. Statistical significance was calculated using Fisher's exact test or chi-square test (significance was supposed when *P* level was < 0.05), respectively. An odds ratio (OR) was calculated if reasonable. Metric data were analyzed using either Student's *t*-test or Welch's test (significance was supposed when *P* level was < 0.05).

## 3. Results

During the period between May 2012 and June 2013 a total of 37 patients (13 female, 24 male, average age 46.6 years, SD 19.7 years) were included in the intervention group and treated according to the SOP. The historical comparison group (“standard care group”) consisted of 109 patients (43 female, 66 male, average age 52.6 years, SD 15.5 years). SAH was the main diagnosis within the standard care group (47 patients). 35 patients suffered from severe traumatic brain injury, 3 from meningoencephalitis, 7 from cerebral ischemia, and 13 from other types of intracranial hemorrhage, and 2 presented with sinus venous thrombosis, 1 with an intracerebral tumor, and 1 with an arteriovenous malformation (AVM). The intervention group consisted of 8 SAH patients, 19 severe brain trauma patients, 8 patients with other types of intracerebral hemorrhage, 1 patient with an intracerebral tumor, and 1 patient with an AVM. In both groups patients suffering from the three main diagnoses accounted for 87–95% of the group volume.

**Figure 1:**
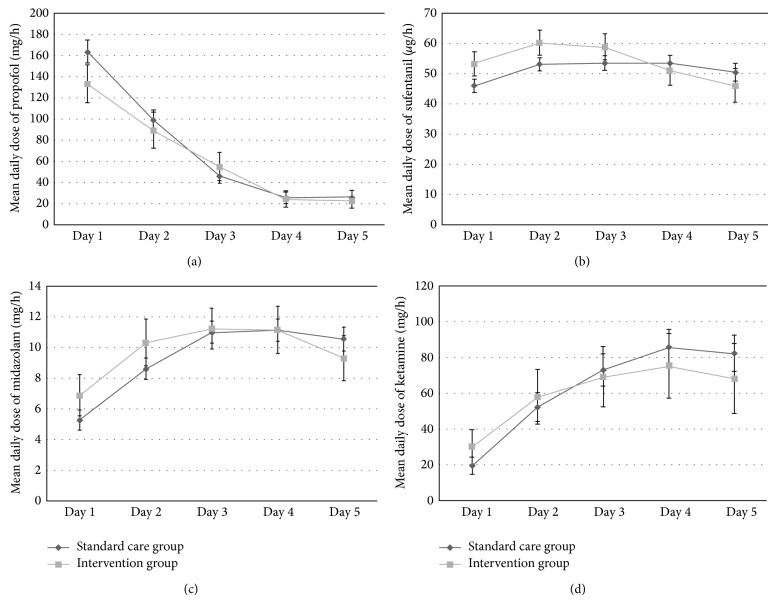
No significant differences in the mean daily dose between the standard care and the intervention group were seen for propofol, sufentanil, midazolam, and ketamine.

**Figure 2:**
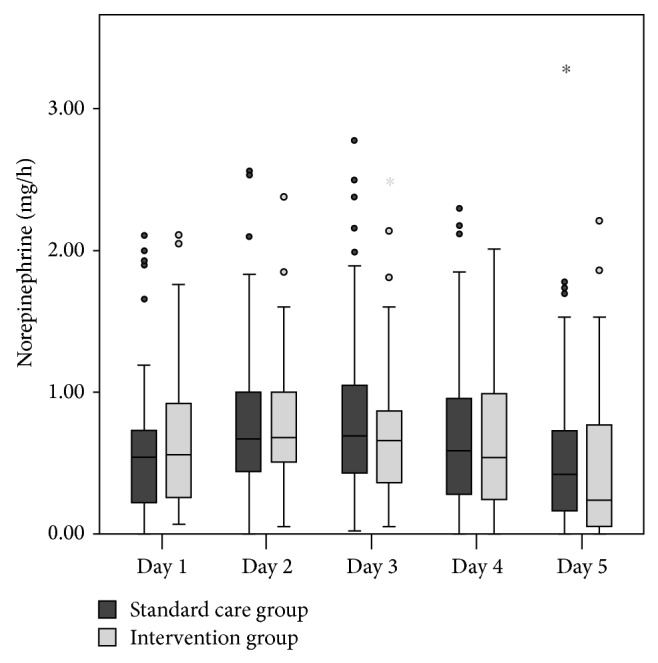
Box plot depicting the mean daily dose of norepinephrine in each of both groups. There was also no significant difference between the two groups.

Propofol, midazolam, and ketamine were given continuously for sedation and sufentanil was used for analgesia. No significant dose differences for each hypnotic as well as the opioid were found between the standard care and the intervention group (Figure 1). The mean daily dose of norepinephrine did not significantly differ between the two groups either (Figure 2).

The main difference between the two groups was found in the defecation pattern. Nine out of 37 (24.3%) patients of the intervention group showed an adequate defecation pattern within the 5-day study period compared to only 9 out of 109 (8.3%) in the standard group. This difference was statistically significant (*P* 0.01, OR 3.6, Table 2). Excessive defecation was equally distributed and had been seen in 2 of 37 patients (5.4%) of the intervention group and in 7 of 109 patients of the standard group (6.4%, *P* 0.59, Table 2).

There was no significant difference in the mean daily value of ICP between the two study groups, although we observed as mentioned an adequate defecation pattern significantly more often in the intervention group (Table 3).

The daily fluid balances (calculation: mean daily parenteral and enteral influx minus mean daily efflux by urine, stool, and other losses) and amounts of given enteral nutrition did not differ between the two groups either during the study period (Tables 4 and 5).

Standard care group patients had to be ventilated mechanically for an average of 14.1 days, whereas intervention group patients only were ventilator-dependent for 12.1 days. This was a mean difference of 2.0 days (95% CI −0.4–4.4) between the groups which, however, did not reach statistical significance (*P* 0.10). The mean length of ICU stay was 24.9 days in the standard care group and 26.1 days in the intervention group, respectively. Despite the 1.1-day shorter ICU stay (95% CI −5.2–2.9) in the standard care group, there was also no statistical significance (*P* 0.58).

Overall, regardless of the group affiliation, 18 patients revealed an adequate defecation pattern. However, ICP values during the first 5 days of ICU stay were comparable in patients with and without adequate defecation. The amount of given enteral nutrition was also comparable between “defecators” and “nondefecators.” Finally there was no significant difference in the duration of mechanical ventilation (14.7 versus 13.5, 95% CI −5.2; 2.7, *P* 0.52) or ICU stay (25.3 versus 25.2 days, 95% CI −6.0; 5.7, *P* 0.97) between patients showing adequate or inappropriate defecation pattern.

## 4. Discussion

In the present study we could demonstrate that the installment of a SOP for promotility drugs and nonpharmacological interventions in analgosedated patients with an acute serious cerebral disorder led to a significantly improved defecation pattern when compared to a historical patient collective. Despite this promising finding we could not show any positive effects of the improved defecation pattern on ICP, length of ICU stay, or ventilator dependency.

SOPs intend to assist in routine daily practice and to guarantee the realization of standards in the critical care setting. Currently, there is a lack of high-grade evidence guidelines dealing with gut motility and laxative regulation in intensive care patients. To improve bowel movement we implemented a SOP bundle consisting of drug ordering sequences and nonpharmacological measures on our neurosurgical ICU. To date only few investigations examined the effectiveness of particular promotility drugs in the critically ill patients [4, 11–14] and were not capable of leading to a conclusive evidence-based strategy. Therefore we based our SOP mainly on theoretical considerations. In contrast to use of only a single treatment we intended to use synergies with step-by-step increasing intensity if defecation was not sufficient. Implementing the SOP into the PDMS provided the option to order the standard treatment bundle automatically at ICU admission for the current and the following days. Automated PDMS-based ordering systems are already established in our and other ICUs, for example, on the field of weaning protocols [15, 16], and are usually followed precisely by the ICU staff. The literature-based prevalence of constipation in critically ill patients varies from 16 to 84% [6–8, 17–19] and from 15.7 to 38% for diarrhea, respectively [8, 17]. Inhomogeneous study populations may explain the broad prevalence spread but the lack of an integrative definition of regular bowel movement or defecation might take the lion's share of the problem; for example, for diarrhea one can find 33 different definitions [20]. Hence we used a study definition of adequate or excessive defecation that, from our point of view, appears reasonable in clinical daily routine. Relating to this definition our study elicited that the implementation of a SOP for the use of promotility drugs and nonpharmacological promotility therapies significantly improved the defecation pattern of neurosurgical ICU patients. However, an excessive amount or frequency of stool was not seen in the intervention group patients. In contrast to our results a recently published study by Knowles et al. did not find an effect of a “bowel management protocol” on the incidence of constipation or diarrhea [21]. The authors presumed an insufficient anchorage of the protocol in daily practice being the main reason for the lacking effect. In our study this “transformation problem” was eliminated by using a highly automated ordering process via PDMS. In addition, the fact that both groups showed no significant difference in dose rates of sedatives and analgetics, especially opioids, as well as vasopressors which are clearly associated with constipation [5, 22], eliminates a potential effect of deeper analgosedation or more intensive vasopressor therapy on the defecation pattern in this study, even if the SOP contained no dose proposals for these topics. In addition, differing fluid balances or amounts of given enteral nutrition as potential influencing factors could also be excluded.

An association between extracranial influences on ICP such as elevated intra-abdominal pressure (IAP) has been repeatedly described [1]. Data from the literature dealing with the development of elevated IAP levels over time in critically ill patients is scarce and inhomogeneous [23–25] but inadequate defecation is one reason for increased IAP. Interestingly we could not establish a positive correlation between improved defecation pattern in the intervention group and ICP values. Despite the findings that prolonged constipation might lead to an elevation in IAP, this is not necessarily the case. Due to the fact that we did not measure IAP we cannot conclude that constipation in our patient collective led to an increase in IAP, thus influencing ICP as described previously [1, 26–29]. In addition, the relatively low mean ICP values in our collectives might represent the fact that the brain autoregulation as well as its mechanisms to compensate for mildly elevated intra-abdominal compartment pressure was still intact. It is also likely that within the first days of ICU stay in patients without serious intra-abdominal disorder the IAP does not rise to a level that might be capable of influencing ICP.

The duration of mechanical ventilation and the length of ICU stay did not differ between the groups. A shortening of mechanical ventilation and ICU stay was described if a regular defecation pattern could be achieved within the first six days after ICU admission [4, 5]. Constipation in contrast was associated with longer ICU stay and a higher weaning failure rate [2, 7]. It is again likely that the chosen investigation period in our study was too short to answer questions concerning the duration of mechanical ventilation or ICU stay sufficiently and length of ventilation and ICU stay depend on various other factors like developing pneumonia or delirium.

Overall our study has some shortcomings. Even if the incidence of adequate defecation pattern was statistically significantly higher in the intervention group only 24.3% of the patients in the intervention group had adequate defecation. This is a relatively low percentage, giving rise to the thought that either the SOP might be insufficient to reach the targeted goal of adequate defecation or our definition was too tight. From a clinical standpoint it would have possibly been sufficient if a patient had shown an adequate defecation on only one day of the whole study period. In addition, the first 5 days after ICU admission might reflect the critical initial phase in brain injury with often the requirement for deep analgosedation and circulatory support but may not reflect the most decisive period for the impact of bowel motility disturbances on ICP and the duration of ICU stay or mechanical ventilation. Thus additional studies using IAP measurement as well as longer observation times are needed to evaluate influence of defecation pattern on IAP and ICP. The latter should also been investigated in patients with higher ICP levels that might have compromised compensation mechanisms.

## 5. Conclusion

Our study showed that a standardized ordering bundle of promotility drugs and nonpharmacological interventions, applied immediately on ICU admission for the first 5 days, is capable of improving defecation pattern in critically ill patients suffering from acute severe brain disorder. However, there was no influence of the SOP on the mean ICP values, the duration of mechanical ventilation, or the length of ICU stay. More studies are necessary to evaluate the effect of bowel movements on IAP and ICP in neurosurgical critically ill patients.

## Figures and Tables

**Table 1:** SOP: promotility procedures for adult patients with acute severe brain disorder and expectable prolonged ventilation for more than 3 days.

	Procedures/promotility drugs
Day 1 = admission day	No procedures/drugs

Day 2	(i) Colon massage + physical therapy (ii) Bisacodyl 10 mg supp. 1x/day (iii) Lactulose 10 mL p.o. 2x/day (iv) Clyster 50 mL 1x/day (v) Sodium picosulfate 10 gtt p.o. 1x/day

Day 3	(i) Colon massage + physical therapy (ii) Lactulose 10 mL p.o. 2x/day (iii) Naloxone 4 mg p.o. 3x/day (iv) Sodium picosulfate 20 gtt p.o. 1x/day (v) Rhizine oil 20 mL p.o. 1x/day

Day 4	(i) Colon massage + physical therapy (ii) Return-flow enema 1x/day (iii) Lactulose 10 mL p.o. 2x/day (iv) Naloxone 4 mg p.o. 3x/day (v) Neostigmine-infusion 1.5 mg 0.25 mg/h i.v. 1x/day

Day 5	(i) Colon massage + physical therapy (ii) Return-flow enema 1x/day (iii) Lactulose 10 mL p.o. 2x/day (iv) Naloxone 4 mg p.o. 3x/day (v) Neostigmine-infusion 1.5 mg 0.25 mg/h i.v. 1x/day

Deescalation after adequate defecation to the following	
(i) Lactulose 10 mL p.o. 2x/day (8:00 a.m. + 8:00 p.m.)	
(ii) For opioid use: naloxone 4 mg p.o. 3x/day (8:00 a.m. + 4:00 p.m. + 12:00 p.m.)	
(iii) Simethicone if necessary 5 mL p.o. 4x/day (2:00 a.m. + 8:00 a.m. + 2:00 p.m. + 8:00 p.m.)	

**Table 2:** Standard care and intervention group. Adequate defecation was observed significantly more often in the intervention group, whereas excessive defecation was seen as frequent in intervention group and in standard care group.

	Total patients *N*	Female *n* (%)	Male *n* (%)	Mean age [years] (SD)	Adequate defecation *n* (%)	Excessive defecation *n* (%)
Standard care group	109	43 (39.4)	66 (60.6)	52.6 (15.5)	9 (8.3)	7 (6.4)
Intervention group	37	13 (35.1)	24 (64.9)	46.6 (19.7)	9 (24.3)	2 (5.4)

					**P 0.01** **OR 3.6**	**P 0.59**

*n*: number, SD: standard deviation, and OR: odds ratio.

**Table 3:** Mean daily ICP values.

	MW Standard care group (mmHg)	MW Intervention group (mmHg)	MD (mmHg)	95% CI	*P* level
Day 1	10.6	10.0	0.6	−1.7; 2.8	0.60
Day 2	11.2	12.0	−0.8	−2.2; 0.6	0.27
Day 3	11.4	11.9	−0.5	−1.9; 0.8	0.43
Day 4	11.3	10.7	0.6	−0.9; 2.0	0.44
Day 5	12.4	10.4	2.0	−0.5; 4.4	0.12

MW: mean value, MD: mean difference, and CI: confidence interval.

**Table 4:** Daily fluid balance.

	MW Standard care group (L)	MW Intervention group (L)	MD (L)	95% CI	P level
Day 1	0.76	0.91	−0.15	−0.73; 0.42	0.59
Day 2	1.20	1.40	−0.20	−0.91; 0.51	0.57
Day 3	0.89	0.87	0.01	−0.42; 0.45	0.95
Day 4	0.46	0.75	−0.29	−0.76; 0.17	0.21
Day 5	0.21	0.33	−0.12	−0.58; 0.33	0.60

MW: mean value, MD: mean difference, and CI: confidence interval.

**Table 5:** Daily amount of given enteral nutrition.

	MW Standard care group (mL)	MW Intervention group (mL)	MD (mL)	95% CI	*P* level
Day 1	21.7	4.3	17.4	−0.1; 34.8	0.05
Day 2	221.2	267.9	−46.7	−145.1; 51.8	0.35
Day 3	484.9	478.1	6.8	−129.2; 142.8	0.92
Day 4	674.8	837.1	−162.3	−348.0; 23.4	0.09
Day 5	790.0	976.4	−186.4	−414.5; 41.8	0.11

MW: mean value, MD: mean difference, and CI: confidence interval.
